# Adjunct low‐dose interleukin‐2, protein nitration and early neurologic changes after acute ischaemic stroke: A pilot observational study

**DOI:** 10.1002/ctm2.70824

**Published:** 2026-09-08

**Authors:** Xue Li, Haiping Zhao, Xiang Lin, Xunming Ji, Jing He

**Affiliations:** ^1^ Department of Rheumatology and Immunology, Peking University People's Hospital; Beijing Key Laboratory of Non‐invasive Diagnosis and Immunotherapy of Rheumatic Diseases (Peking University People's Hospital) Beijing China; ^2^ Neuro‐Cardio‐Vascular Diseases Research Lab Xuanwu Hospital of Capital Medical University Beijing China; ^3^ School of Chinese Medicine Li Ka Shing Faculty of Medicine The University of Hong Kong Hong Kong SAR China; ^4^ Chinese Academy of Medical Sciences (CAMS) Peking Union Medical College (PUMC) Beijing China

Dear Editor,

Protein nitration, reflected by 3‐nitrotyrosine (3‐NT) levels, contributes to oxidative and inflammatory injury after cerebral ischaemia and may contribute to neurologic damage after acute ischaemic stroke (AIS).^1^ Studies have implicated nitrative stress in post‐ischaemic neuronal injury, and elevated 3‐NT has been reported in patients with AIS. However, whether immune modulation is associated with changes in protein nitration in AIS remains unknown.^2^, ^3^ Low‐dose interleukin‐2 (IL‐2) preferentially increases regulatory T cells (Tregs) populations and has shown immunomodulatory effects in patients with AIS. However, whether IL‐2 is associated with changes in protein nitration in AIS remains unknown. We therefore evaluated peripheral‐blood mononuclear cell (PBMC) 3‐NT levels in patients with AIS receiving adjunct low‐dose IL‐2.

We conducted an exploratory, nonrandomized, unblinded three‐group observational study (*n* = 6 per group) with assessments at baseline and Day 6 of treatment; no patient underwent thrombolysis or endovascular therapy. Participants received treatment according to routine clinical practice and their individual treatment preferences, rather than through random assignment. Patients with AIS received standard therapy including edaravone, with or without adjunct low‐dose interleukin‐2 (1 × 10^6^ IU, administered subcutaneously on alternate days).^4^ Baseline demographic, clinical and immunologic measures did not differ significantly between the two stroke groups. (Table 1). A connective‐tissue disease group receiving low‐dose IL‐2 alone served as a biologic comparator to explore whether changes in 3‐NT might represent a broader biologic effect of IL‐2. Neurologic severity was assessed with the National Institutes of Health Stroke Scale (NIHSS). Circulating Tregs were quantified by flow cytometry,^5^ whereas peripheral‐blood mononuclear cells (PBMCs) protein nitration was evaluated at baseline and day 6 by western‐blot analysis of total 3‐NT.^6^, ^7^ Because this was an exploratory pilot study, the initial sample size was not determined by a formal prospective power analysis; instead, the study aimed to obtain preliminary estimates of clinical and biologic changes. To inform the design of a subsequent confirmatory study, the required sample size was estimated from the between‐group difference Δ3‐NT in PBMC observed in the present cohort. With a two‐sided α of 0.05 and 90% statistical power, the estimated enrolment was 12 participants in each group; after accounting for a 20% dropout rate, the target was increased to 15 participants per group.

**TABLE 1:** Baseline characteristics of the participants.

Characteristics	Edaravone (*n = 6*)	Ld‐IL2 + Edaravone (*n = 6*)	*p* value
Age, median (IQR), year	70.00 (63.25 to 72.25)	68.50 (64.25 to 74.25)	.9524
Female, No. (%)	2 (33.33)	3 (50.00)	–
**Immune Cell Subsets, median (IQR)**			
Regulatory T cell% in CD4+T	4.22 (3.75 to 4.44)	4.58 (4.34 to 5.16)	.3939
**Nervous System Data, median (IQR)**			
NIHSS Score	4.50 (4.00 to 5.75)	5.00 (4.25 to 6.50)	.6732
**Comorbidity Data, No. (%)**			
Hypertension	6 (100.00)	4 (66.67)	–
Diabetes mellitus	1 (16.67)	3 (50.00)	–
Dyslipidaemia	5 (83.33)	4 (66.67)	–
Coronary artery disease	2 (33.33)	1 (16.67)	–

In the IL‐2–plus–edaravone group, NIHSS scores decreased from 5.00 (4.25 to 6.50) at baseline to 3.50 (3.00 to 4.75) on Day 6 (*p* < .05) (Figure 1B). In contrast, patients treated with edaravone‐alone showed a smaller reduction, with NIHSS scores changing from 4.50 (4.00 to 5.75) to 4.00 (2.50 to 4.75) over the same period (*p* = .1250) (Figure 1A). When comparing the magnitude of neurological improvement between groups, the reduction in NIHSS score (ΔNIHSS) was numerically greater in the IL‐2–plus–edaravone group [−1.50 (−2.00 to −1.00)] than in the edaravone‐alone group [−1.00 (−1.00 to −.25)] (*p* = .2316) (Figure 1B). Although the between‐group difference was not statistically significant.

**FIGURE 1:**
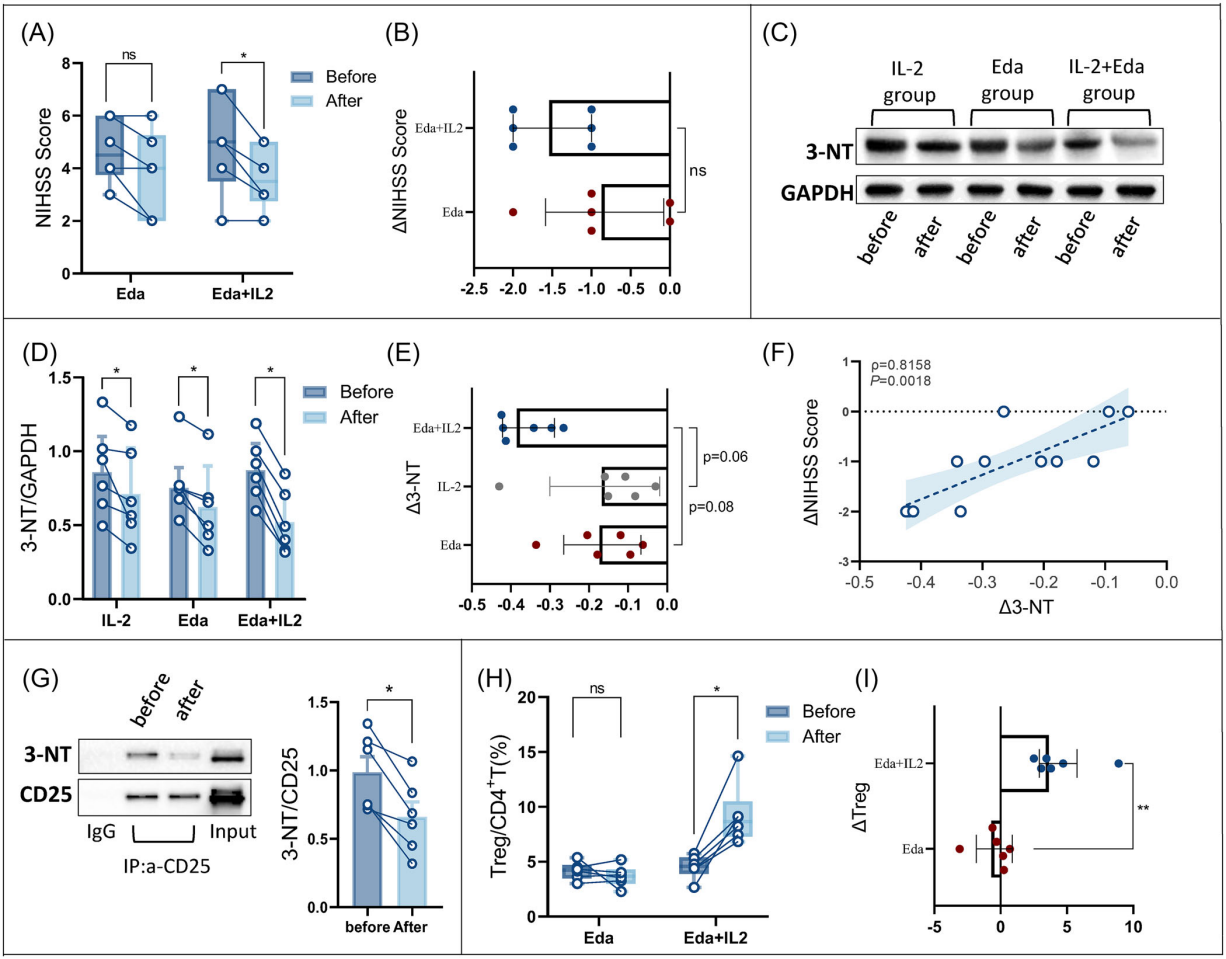
Neurologic improvement, protein nitration and immune changes with low‐dose il‐2 in acute ischaemic stroke. (A and B) NIHSS scores at baseline and Day 6 and the change from baseline to Day 6 (ΔNIHSS) in the edaravone‐alone group and the IL‐2–plus–edaravone group (*n *= 6 per group). (C–E) Protein expression and quantification of total 3‐nitrotyrosine (3‐NT) in peripheral‐blood mononuclear cells (PBMCs) at baseline and Day 6 and the change from baseline to Day 6 (Δ3‐NT) (*n* = 6 per group). (F) Association between ΔNIHSS and Δ3‐NT (Spearman correlation; *n* = 12). (G) CD25 (IL‐2Rα) nitration in paired peripheral‐blood mononuclear cell samples from the IL‐2–plus–edaravone group (*n* = 6), assessed by CD25 immunoprecipitation followed by 3‐nitrotyrosine immunoblotting, with quantification of the 3‐NT/CD25 ratio before and after treatment. (H and I) Percentage of circulating regulatory T cells (Tregs) at baseline and Day 6 and the change from baseline to Day 6 (ΔTreg%) in the edaravone‐alone group and the IL‐2–plus–edaravone group (*n *= 6 per group). Data are medians and interquartile range. Δ denotes Day 6 minus baseline. Between‐group comparisons were performed with the Mann–Whitney *U* test, within‐group comparisons with the Wilcoxon signed‐rank test, and three‐group comparisons with the Kruskal–Wallis test followed by Dunn's multiple‐comparisons test. Correlations were assessed with Spearman's rank correlation. *p < .05, **p < .01.

Within‐group analyses showed significant reductions in PBMC 3‐NT levels from baseline to Day 6 in all three groups [Edaravone‐alone group  .71 (.61 to  .76) vs.  .58 (.38 to  .90), *p* < .05; IL‐2–plus–edaravone group:  .87 (.78 to  .96) vs.  .45 (.33 to  .60), *p* < .05; connective‐tissue disease treated with IL‐2 alone group:  .71 (.57 to  .98) vs.  .62 (.43 to 1.08), p < .05] (Figure 1 C, D). Total 3‐NT levels in PBMCs decreased more in the IL‐2–plus–edaravone group [Δ, −.38 (−.42 to −.28)] than in the edaravone‐alone group [Δ, −.15 (−.27 to −.08)]. A directional reduction in 3‐NT was also observed in connective‐tissue disease treated with IL‐2 alone [Δ, −.13 (−.29 to −.06)] (Figure 1E). ΔNIHSS score correlated with Δ3‐NT levels (Spearman ρ = .82, *p* < .05) (Figure 1F). In an exploratory analysis, CD25 was immunoprecipitated from PBMC lysates and subsequently immunoblotted for 3‐NT. In the IL‐2–plus–edaravone group, the 3‐NT/CD25 ratio decreased from  .95 (.73 to 1.18) at baseline to  .53 (.35,  .80) on day 6 (*p* < .05), showing reduced CD25‐associated nitration after treatment (Figure 1G). In parallel, circulating Tregs increased significantly in the combination group [4.58 (4.34 to 5.16) vs. 7.84 (7.38 to 8.87), *p* < .05, Δ, 3.62 (3.15, 4.48)], whereas changes were less pronounced in the edaravone‐alone group (Figure 1H,I).

A reduction in PBMC 3‐NT was also observed in the connective‐tissue disease cohort receiving low‐dose IL‐2, providing supportive biologic context; however, differences in disease background preclude direct cross‐disease inference. Given the small sample size, short treatment and observational design, these results should be considered hypothesis‐generating. Importantly, treatment allocation was nonrandomized and unblinded, introducing substantial potential for selection bias and residual confounding. Accordingly, the observed between‐group differences cannot establish a causal therapeutic effect of low‐dose IL‐2. Larger randomized trials are warranted to characterize the temporal changes in 3‐NT, regulatory T cells, and neurologic outcomes, and to determine whether 3‐NT may serve as a pharmacodynamic biomarker of IL‐2 activity and whether these biologic changes translate into clinical benefit.

## CONCLUSION

Adjunct low‐dose IL‐2 was associated with greater reductions in PBMC protein nitration and increased circulating Tregs, together with numerically greater NIHSS improvement in AIS. Changes in 3‐NT correlated with changes in NIHSS, supporting further investigation of protein nitration as a biologic response to low‐dose IL‐2.

## AUTHOR CONTRIBUTIONS

Xue Li collected the clinical data, performed the statistical analyses and data visualization, conducted flow cytometry and Western blotting on patients’ peripheral blood samples, interpreted the data, prepared the figures, drafted the manuscript and coordinated revisions. Haiping Zhao participated in data interpretation, acquired funding and critically reviewed and revised the manuscript. Xiang Lin contributed to data collection and analysis, acquired funding and critically reviewed and revised the manuscript. Xunming Ji supervised the study, provided scientific guidance throughout the project and critically reviewed and revised the manuscript. Jing He conceived and designed the study, supervised the overall project, contributed to data analysis and interpretation, acquired funding, and critically reviewed and revised the manuscript. All authors read and approved the final manuscript.

## CONFLICT OF INTEREST STATEMENT

The authors declare no conflicts of interest.

## FUNDING INFORMATION

This study was supported by grants from the International Science and Technology Cooperation Program (Hong Kong, Macao, and Taiwan) (grant no.: Z251100007125012) and the National Natural Science Foundation of China (grant no.: 82271309).

## CONSENT FOR PUBLICATION

Not applicable.

## ETHICS APPROVAL

The study protocol received approval from the Peking University People's Hospital Ethics Committee (Approval number: 2020PHB359‐01).

## Data Availability

Data are not publicly available because of participant privacy considerations and the small sample size but may be available from the corresponding author upon reasonable request.
